# Softwaregestützte Analyse geriatrischer Entlassbriefe: Potenziale technologiegestützter Datenanalysen im Qualitäts- und Entlassmanagement am Übergang in die ambulante Versorgung

**DOI:** 10.1007/s00391-025-02478-6

**Published:** 2025-08-18

**Authors:** T. Herzig, C. Marschner, C. Ostrau, S. Held, J. Rickermann, W. Schenck, A. Uphaus, R. Amelung

**Affiliations:** 1 Hochschule Bielefeld, Kurt-Schuhmacher-Str. 6, 33615 Bielefeld, Deutschland; 2 Katholische Hospitalvereinigung Ostwestfalen gGmbH, Bielefeld, Deutschland; 3 Ambulante Geriatrische Rehabilitation Bielefeld GmbH & Co. KG, Bielefeld, Deutschland

**Keywords:** Entlassmanagement, Geriatrie, Transdisziplinarität, Technologie, Datenanalyse, Discharge management, Geriatrics, Transdisciplinarity, Technology, Data analysis

## Abstract

**Hintergrund:**

Im Bielefelder Reallabor Geriatrie der Hochschule Bielefeld wurde in Kooperation mit der Katholischen Hospitalvereinigung Ostwestfalen ein Entlassbriefanalyzer entwickelt. Dieser kann bei der Qualitätssicherung im Bereich des Entlassmanagements unterstützen.

**Methodik:**

Der Entlassbriefanalyzer nutzt Methoden des „Text Mining“, um Informationen in den jeweiligen Entlassbriefen zu finden. Es werden dabei Daten aus dem Freitext als auch Zahlenwerte der dokumentierten Assessments extrahiert. Eine Zuordnung der Fälle zu Diagnosekategorien erfolgte im Projekt sowohl durch eine deduktive als auch induktive Kategorienentwicklung und die Sammlung von Schlagworten durch das ärztlich eingebundene Fachpersonal.

**Ergebnisse:**

Mithilfe des Analyzers konnten Patient*innen unterschiedlichen Diagnosekategorien zugeordnet werden. Es zeigt sich, dass das Gros der Fälle aufgrund multifaktorieller oder sturzbedingter Indikationen behandelt wurde. Zudem verbesserten sich die Patient*innen in verschiedenen Tests über die Zeit der Behandlung. Die meisten Patient*innen konnten nach der Behandlung zurück in die Häuslichkeit entlassen werden.

**Diskussion:**

Anhand der Ergebnisse lässt sich für das Entlassmanagement erkennen, dass es in der anschließenden Behandlung einen besonderen Fokus auf sturzbedingte und neurodegenerative Erkrankungen braucht. Aufgrund der überwiegenden Entlassung der Patient*innen in ihre Häuslichkeit ergibt sich ein wesentliches Augenmerk auf die ambulante Versorgung. Grundsätzlich kann dabei auf eine messbare Verbesserung durch die stationäre Behandlung aufgebaut werden.

**Zusatzmaterial online:**

Zusätzliche Informationen sind in der Online-Version dieses Artikels (10.1007/s00391-025-02478-6) enthalten.

## Abstract (en)

**Background:**

A discharge letter analyzer was developed in the Bielefeld Geriatrics Living Laboratory at Bielefeld University of Applied Sciences in cooperation with the Catholic Hospital Association East Westphalia (Katholische Hospitalvereinigung Ostwestfalen). This can support quality assurance in the area of discharge management.

**Methods:**

The discharge letter analyzer uses text mining methods to find information in the respective discharge letters. Data are extracted from the free text as well as numerical values from the documented assessments. In the project the cases were assigned to diagnostic categories through both deductive and inductive category development and the collection of keywords by the medical staff involved.

**Results:**

With the help of the analyzer patients could be assigned to different diagnostic categories. It was found that the majority of cases were treated due to multifactorial or fall-related indications. In addition, the patients improved in various tests over the course of treatment. Most patients could be discharged back home after treatment.

**Conclusion:**

The results show that discharge management requires a special focus on fall-related and neurodegenerative diseases in the subsequent treatment. Due to the predominant discharge of patients to their homes, there is a significant focus on outpatient care. In principle, a measurable improvement can be achieved through inpatient treatment.

Die Qualitätssicherung im Bereich des Entlassmanagements geriatrischer Patient*innen gewinnt aufgrund demografischer Veränderungen weiter an Bedeutung. Dabei sind Fragen nach den häufigsten Indikationen eines stationären Aufenthalts sowie etwaigen Behandlungsmustern ebenso von Interesse wie Fragen nach der Allokation der Patient*innen sowie einer sich anschließenden ambulanten oder mobilen Rehabilitation. Die Extraktion dieser Daten aus Entlassbriefen akutstationär versorgter Patient*innen kann dabei mittels eines eigens konzipierten Entlassbriefanalyzers automatisiert erfolgen und ermöglicht eine Zuordnung von Patient*innen in vorab definierte Diagnosekategorien.

## Hintergrund und Fragestellung

Das Bielefelder Reallabor Geriatrie ist ein Experimentierraum im Verbund von Hochschule und Versorgungsanbietern der Region Ostwestfalen-Lippe, in dem verschiedene Ansätze der Patientenversorgung unter realen Bedingungen erprobt werden. Ziel dieser Kooperationen ist die Realisierung eines inter- und transdisziplinären Forschungs- und Entwicklungsansatzes, um die medizinische, pflegerische, therapeutische sowie technologische (Gesundheits‑)Versorgung im Bereich der Anschlussheilbehandlung älterer Menschen zu verbessern [4]. In diesem Kontext wurde schließlich eine Möglichkeit entwickelt, Patientendaten sowohl im Sinne des Qualitätsmanagements als auch der Informationsgewinnung für den ambulanten Rehabilitationsbereich durch den im Folgenden näher beschriebenen Entlassbriefanalyzer zu extrahieren.

Auf Grundlage dieser technologischen Möglichkeit, der erwarteten Einsatzpotenziale des Entlassbriefanalyzers und einer bestehenden Kooperation zwischen der Katholischen Hospitalvereinigung Ostwestfalen gGmbH (KHO) mit der Hochschule Bielefeld (HSBI) wurde im Oktober 2023 ein Projekt zur Qualitätssicherung im Bereich des Entlassmanagements geriatrischer Patient*innen initiiert.

Ziel war es, Entlassbriefe der geriatrischen Station des Franziskus Hospital Bielefeld (eines von insgesamt 6 Krankenhäusern der KHO) im Projektverlauf eine datenschutzrechtlich konforme Analyse durchlaufen zu lassen, um etwaige Erkenntnisse über mögliche Verbesserungspotenziale und Impulse für das krankenhausseitige Entlassmanagement zu gewinnen. So sollten sowohl Assessmentergebnisse als auch die empfohlenen Allokationen in den Blick genommen werden. Darüber hinaus wurde der Frage nachgegangen, ob Patientenfälle anhand festgelegter intelligibel erstellter Kriterien in sog. Diagnosekategorien eingeordnet werden können, um darauf aufbauend eine qualitätsgesicherte, optimale Einsatzplanung für die anschließenden Behandlungspfade zu ermöglichen.

## Studiendesign und Untersuchungsmethode

Alle im Folgenden beschriebenen Untersuchungen wurden mit Zustimmung der Deutschen Gesellschaft für Pflegewissenschaft e. V. nach ethischem Clearing am 08.05.2024 und im Einklang mit nationalem Recht gemäß der Deklaration von Helsinki in ihrer letzten Revision von 2013 durchgeführt. Die Analyse der Entlassbriefe erfolgte im Klinikum selbst.

### Praktische Umsetzung und extrahierte Daten

**Abb. 1:**
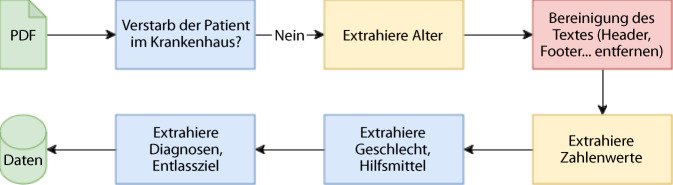
Ablauf der Datenextraktion aus Entlassbriefen. *Blau*: Prozesse, hauptsächlich mit Schlagwortsuche; *gelb*: Prozesse zusätzlich mit Zahlenextraktion

Die Entlassbriefe der Akutgeriatrie im Franziskus-Hospital lagen zunächst als PDF vor und mussten vom Entlassbriefanalyzer eingelesen werden. Anschließend wurde mittels Schlagwortsuche überprüft, ob der/die Patient*in im Krankenhaus verstorben war. Via Textsuche wurde das Dokument nach dem Alter durchsucht und dieses mittels Zahlenextraktion ausgelesen. Das Alter konnte dabei entweder direkt angegeben sein (Alter: xx Jahre) oder mittels Differenz zwischen Geburtsjahr und Behandlungsjahr berechnet werden. Da in allen Fällen das Geburtsjahr stets in der Form „* xx.xx.xxxx“ vorlag, war eine einfache Extraktion möglich. Erst im Anschluss erfolgte die Bereinigung des Dokuments, wobei Kopf- und Fußzeilen entfernt wurden. Zahlenwerte (z. B. beim Barthel-Index) wurden mittels Schlagwortsuche im Dokument und anschließender Zahlenextraktion ermittelt. Das Geschlecht wurde mit Schlagwortsuche (Patientin/Patient) bestimmen, wobei diese auf binäre Geschlechter beschränkt war. Verwendete Hilfsmittel, wie beispielsweise Rollator oder Gehstützen, wurden ebenfalls per Schlagwortsuche im Text gefunden. Diese wurden in den vorliegenden Briefen, durch Kommata getrennt, aufgelistet und entsprechend extrahiert. Im Anschluss wurden Diagnosen durch eine Schlagwortsuche extrahiert. Weiterhin wurden die Empfehlungen zur Allokation von Patient*innen ausgelesen. Eine Übersicht aller erhobenen Parameter findet sich im Online-Supplement: Tab. 1. Abb. 1 veranschaulicht diesen Prozess zur „information extraction“.

Für die Verschlagwortung der „Diagnosekategorien“ wurde zunächst deduktiv eine Liste von Schlag- und Teilworten erstellt. In einem weiteren Schritt wurden zufällige Diagnosen aus den Entlassbriefen ausgewählt und diese mit den deduktiv erstellten Kategorien abgeglichen. Auf Basis dessen wurde die Liste, sofern notwendig, erweitert. Die schlussendlich für die Auswertung verwendeten Begriffe sind im Zusatzmaterial online dargestellt.

### Technisches Vorgehen

Die Extraktion von Informationen aus Freitexten, wie sie in Entlassbriefen in der Regel vorzufinden sind, erfolgte im Projekt mittels Methoden des „Text Mining“ [5] – dem Gewinnen von Informationen aus Volltexten. In dem hiermit vorgestellten Projekt, in dem je Patient*in eine Textdatei zur Verfügung stand, kann grundsätzlich auch von „information extraction“ gesprochen werden [2], welches eine Unterkategorie des Text Mining bildet. Auch wurde sich bewusst gegen die Nutzung von Methoden aus dem Bereich des maschinellen Lernens entschieden: Die für spezialisierte KI Modelle benötigten Trainingsdaten dürfen aus datenschutzrechtlichen Gründen nicht das Krankenhaus verlassen. Im Krankenhaus sind aber nur beschränkte Rechenressourcen verfügbar, sodass ein Training solcher Modelle ausgeschlossen war. Generalisierte Modelle, wie z. B. große Sprachmodelle, sind ihrerseits mit hohem Rechenaufwand verbunden oder extern gehostet, was wieder die Datenschutzproblematik aufwirft. Unser Ziel war es also, mit möglichste einfachen Methoden Informationen aus den Briefen zu extrahieren. Die meisten der im Projekt extrahierten Parameter lagen in der Praxis in halbstrukturierter Form (vergleichbarer Aufbau der Textblöcke) in den Entlassbriefen vor. So wurden beispielsweise Metriken in der Form „Barthel-Index: xx/100“ dokumentiert und ließen sich in dieser Form auch ohne maschinelle Lernverfahren verlässlich auslesen. Diese Extraktionspipeline setzte sich im Projekt aus 2 Kernkomponenten zusammen, der *Schlagwortsuche* und der *Zahlenextraktion*:

Bei der *Schlagwortsuche* wurde jede Zeile des Entlassbriefes nach einem Schlagwort durchsucht. Um Rechtschreibfehler zu berücksichtigen, kam die Levenshtein-Distanz zum Einsatz [7]. Dabei wird die Ähnlichkeit zweier Worte (Satzfragmente) über die Anzahl der abweichenden Stellen berechnet. Zwei Worte gelten als gleich, sofern ein empirisch bestimmter Schwellwert nicht überschritten wird. Die Schlagwortsuche erfolgte ebenfalls zeilenweise, sodass alle Zeilen, die das entsprechende Schlagwort enthielten, im Anschluss weiterverarbeitet werden konnten.

Für die *Zahlenextraktion* werden hingegen reguläre Ausdrücke genutzt: Sofern beispielsweise ein Barthel-Index in der Form xx/100 vorlag, konnte eine Analyse der Zeilen aus der Schlagwortsuche nach diesem Muster erfolgen, und die passenden Werte konnten extrahiert werden. Neben diesem Muster wurde für jeden Zahlenindex ein Intervall hinterlegt, innerhalb dessen valide Zahlenwerte liegen durften. Auf diese Weise konnten fehlerhafte Zahlenwerte aussortiert werden.

Die grundsätzliche Implementierung erfolgte in der Programmiersprache „Python“ und nutzte neben der Standardbibliotheken zusätzlich die Bibliotheken „thefuzz“ und „pandas“.

Die Analyse eines einzelnen Briefes benötigte wenige Sekunden auf einem modernen, herkömmlichen Laptop und war daher mit einem geringen Ressourcenverbrauch verbunden. Im Gegensatz zu anspruchsvollen KI-Methoden erlaubte diese Art der Umsetzung den Einsatz beim Projektpartner. Hochsensible Patientendaten verließen das Krankenhaus somit nicht. Die extrahierten Daten ermöglichten keinen Rückschluss auf einzelne Personen. Die Grundlage für die Verarbeitung war die Einwilligung der Patient*innen in die Verarbeitung zur Qualitätssicherung. Gleichwohl konnten nicht aus allen 372 Briefen alle erhobenen Parameter extrahiert werden, da Assessments z. T. nicht durchgeführt oder fehlerhaft übertragen wurden.

## Ergebnisse

Mithilfe des Entlassbriefanalyzers konnten nach Ausschluss von 21 Dubletten die benannten 372 Entlassbriefe ausgewertet werden. Anhand der analysierten Briefe ließen sich 274 Patientinnen und 98 Patienten identifizieren. Das Alter betrug durchschnittlich 84,4 ± 6,6 Jahre. Die Ergebnisse lassen sich in 3 Analysebereiche differenzieren: „Analyse der Diagnosekategorien“, „Auswertung von Assessmentergebnissen“ sowie „Allokation“ einteilen:

### Analyse der Diagnosekategorien

**Abb. 2:**
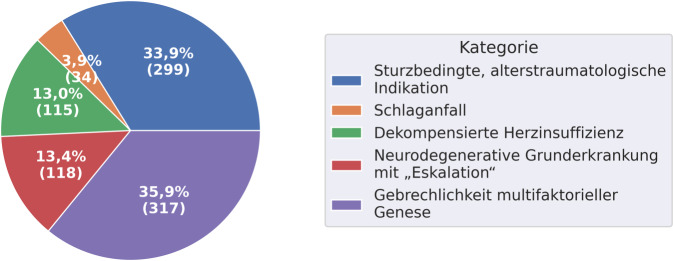
Zuordnung zu den Diagnosekategorien. Mehrfachzuordnung möglich

Bei reiner Betrachtung der Diagnosekategorien (Abb. 2), ist zu erkennen, dass sich 35,9 % der Patient*innen mit „Gebrechlichkeit multifaktorieller Genese“ in der stationär-geriatrischen Behandlung befinden. 33,9 % konnten den „sturzbedingten, alterstraumatologischen Indikationen“ zugeordnet werden. Zu 13,4 % haben Patient*innen eine „neurodegenerative Grunderkrankung mit Eskalation“ und zu 13 % eine „dekompensierte Herzinsuffizienz“. Symptome der Diagnosegruppe „Schlaganfall“ wiesen in der Stichprobe 3,9 % aller Patient*innen auf.

**Abb. 3:**
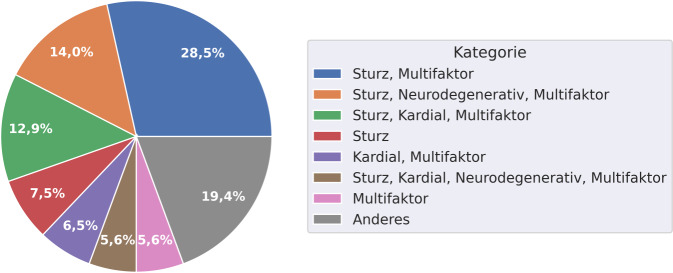
Häufigste Cluster der Diagnosekategorien. Kategorien wie folgt abgekürzt: *Sturz*bedingte, alterstraumatologische Indikation; *Schlaganfall*; dekompensierte Herzinsuffizienz (*kardial)*, *neurodegenerativ*e Grunderkrankung mit „Eskalation“; Gebrechlichkeit *multifaktor*ieller Genese

Bei einer Clusterung der Diagnosekategorien, die in Abb. 3 zu erkennen ist, kann festgestellt werden, dass 28,5 % der Patient*innen eine Kombination von „sturzbedingte, alterstraumatologische Indikation“ und „Gebrechlichkeit multifaktorieller Genese“ aufweisen. Mit 14,0 % stellt die Kombination aus „sturzbedingte, alterstraumatologische Indikation“, „neurodegenerative Grunderkrankung mit ‚Eskalation‘“ und „Gebrechlichkeit multifaktorieller Genese“ die zweitgrößte Kombinationsgruppe dar. Zusammen mit dem Cluster „sturzbedingte, alterstraumatologische Indikation“, „dekompensierte Herzinsuffizienz“, „Gebrechlichkeit multifaktorieller Genese“ (12,9 %) bilden diese über die Hälfte der analysierten Fälle ab.

### Auswertung von Assessmentergebnissen

**Abb. 4:**
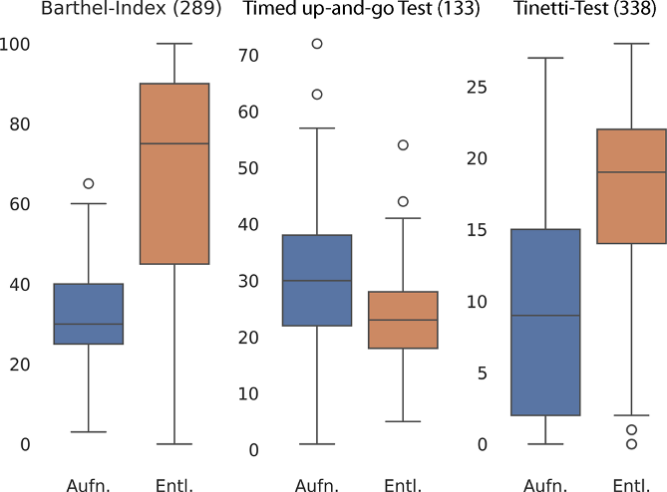
Boxplot für 3 Indikatoren der geriatrischen Behandlung

**Abb. 5:**
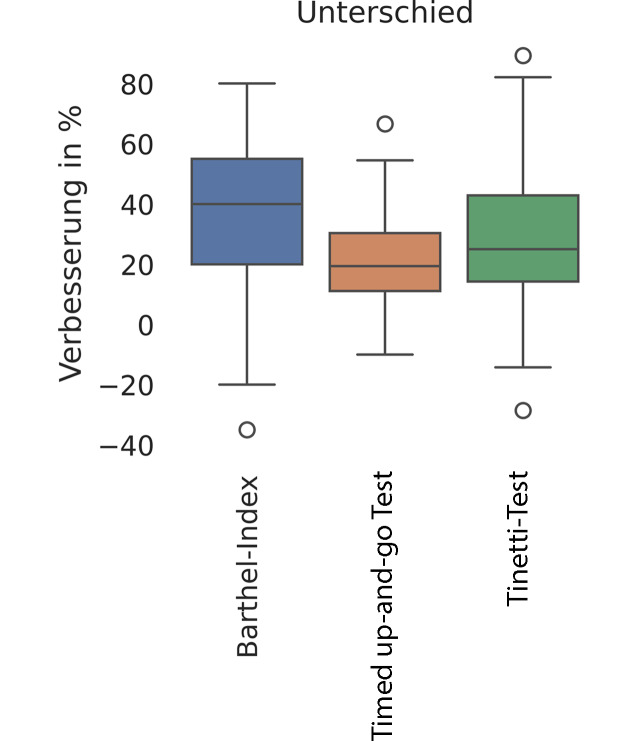
Patientenweise Verbesserung der 3 Indikatoren durch stationären Aufenthalt

Im Entlassbrief werden die Resultate diagnostischer Testverfahren dokumentiert. In der vorliegenden Auswertung wird der Fokus auf den „Tinetti-Test“, den „Barthel-Index“ und den „Timed up-and-go Test“ gelegt, da diese Testverfahren bei der Aufnahme sowie bei der Entlassung der Patienten in der überwiegenden Anzahl der Fälle durchgeführt wurden. Bei anderen Tests, wie dem „Uhrenergänzungstest“, wurden die Stichproben häufig nur einmal erhoben und auch nicht bei allen Patienten, sodass die Stichprobengröße in diesen Fällen deutlich geringer ausfällt. Die Daten für die zuvor genannten Tests konnten mit dem Entlassbriefanalyzer ausgelesen und analysiert werden. Dies ermöglicht die Ableitung der folgenden Erkenntnisse bezüglich des Potenzials der Versorgung:

In Abb. 4 werden zunächst die Boxplots der einzelnen Assessments aufgezeigt. Die Tendenz ist in allen Tests eindeutig in Richtung einer Verbesserung. Die Grafik der Unterschiede in Abb. 5 verdeutlicht diese Tendenz ebenfalls. Die Darstellung der Ergebnisse ist relativ und bezieht sich auf die Verbesserung in den jeweiligen Skalen der einzelnen Tests. Die Verbesserung beträgt je nach Test im Median 40 % (Barthel-Index), 20 % (Timed up-and-go Test) und 25 % (Tinetti-Test). Der Großteil der Patient*innen erfährt eine Verbesserung. Es zeigt allerdings auch, dass dies nicht für alle Patient*innen gilt. Manche Patient*innen erleben im Vergleich zur Eingangsuntersuchung eine Verschlechterung in den Testergebnissen um bis zu 35 % (s. Ausreißer „Barthel-Index“). Dennoch ist auf die allgemeine positive Tendenz, die im anderen Extrem auch eine Verbesserung um bis zu 80 % bedeuten kann, eindeutig erkennbar.

### Allokation

Patient*innen, die aus der akutgeriatrischen Komplexbehandlung entlassen werden, wird in der Regel eine anschließende Behandlung empfohlen. Eine Durchführung dieser ist damit zwar noch nicht gewährleistet, es lässt sich dennoch ableiten, welche Versorgungsform Patient*innen nach einer stationären geriatrischen Rehabilitation erwarten könn(t)en oder müss(t)en.

**Abb. 6:**
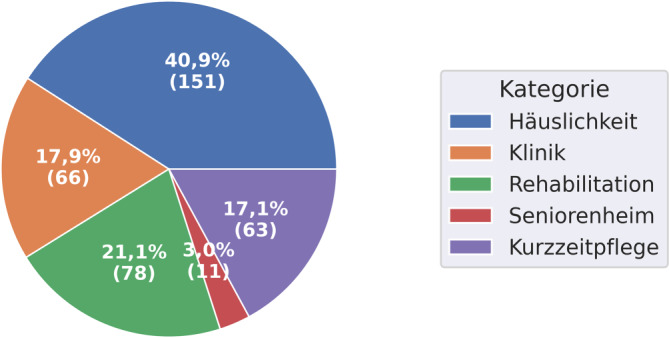
Aufschlüsselung der verschiedenen poststationären Allokationen

Wie Abb. 6 zeigt, wird in 40,9 % der Fälle die Häuslichkeit als poststationäre Allokation implizit oder explizit benannt. 17,9 % der Personen werden im Anschluss hingegen in eine (andere) Klinik entlassen. In 21,1 % schließt sich eine Rehabilitation an. Mit 3,0 % geht ein geringer Anteil der Patient*innen ins Seniorenheim, wobei in keinem Entlassbrief das betreute Wohnen als anschließende Wohnform vorgeschlagen wird. Nicht ersichtlich ist dabei, inwieweit die eigene Häuslichkeit ggf. eine Form des betreuten Wohnens sein könnte. In 17,1 % der Fälle schließt sich die Kurzzeitpflege der stationären Rehabilitation an. Was im Anschluss mit den Patient*innen geschieht, konnte aufgrund der Methodik nicht konkreter analysiert werden.

## Diskussion und Methodenkritik

Die Versorgung geriatrischer Patient*innen ist herausfordernd und begründet aufgrund des steigenden Bedarfs an Versorgungskapazitäten [1, 3] und einer Zunahme der Multimorbidität v. a. bei dieser Klientel [1] die Intensivierung der Forschung – auch hinsichtlich des Umgangs mit Daten im Sinne des Qualitätsmanagements.

Die Einteilung in Diagnosekategorien kann eine Grundlage darstellen, um im Sinne des Qualitäts- und Entlassmanagements die Bedarfe geriatrischer Patient*innen in anschließenden Versorgungsformen zielgerichteter zu klären. Im Rahmen dieser Erhebung konnten lediglich Daten aus einer akutgeriatrischen Klinik genutzt werden, was die Generalisierbarkeit der Aussagen einschränkt. Grundsätzlich könnte auf der Basis der entwickelten Diagnosekategorien das Team einer ambulanten geriatrischen Rehabilitation zusammengesetzt und eine Einsatzplanung für anschließende Behandlungspfade erstellt werden. Hierzu müsste allerdings die gesamte Patientenklientel dieser ambulanten Einrichtung bekannt sein. In dem konkreten Fall könnte begründet werden, dass ein Fokus auf die Themen Sturz und Neurodegeneration sinnvoll erscheint, zumal diese beiden Themen auch in der Clusterung der Diagnosekategorien in unserer Stichprobe sehr häufig vorkamen und sich auch aus dem klinischen Bild neurodegenerativer Erkrankungen ergeben. Darüber hinaus könnten gleichzeitig die akuten zerebralen Erkrankungen wie Schlaganfall adressiert werden, zumal auch in diesem Krankheitsspektrum häufig Stürze und die Gehfähigkeit eine zentrale Rolle für die Unabhängigkeit und Teilhabe im täglichen Leben spielen. Die Studienergebnisse der analysierten Patientenklientel verdeutlichen zugleich, dass sich aufgrund der am häufigsten auftretenden Diagnosekategorie der multifaktoriellen Gebrechlichkeit, die auch in der Clusterung der Diagnosekategorien am häufigsten auftrat, und der ebenfalls häufigen kardialen Erkrankungen, ein breit gefächerter geriatrischer Versorgungsbedarf ergibt. Somit könnte für unsere Stichprobe zwar eine Schwerpunktsetzung auf die Themen „Sturz“ und „Neurodegeneration/neurologisches Defizit“ sinnvoll sein, es sollte aber gleichzeitig die Breite der Bedarfe abgedeckt werden können. Um eine Generalisierbarkeit der in dieser Stichprobe gewonnenen Erkenntnisse zu ermöglichen, wäre eine Analyse weiterer Stichproben aus anderen akutgeriatrischen Kliniken erforderlich. Grundsätzlich erscheint eine lokale Analyse der Klientel der zuweisenden Kliniken und ambulanten Behandlern im Einzugsgebiet für eine zielgenaue Planung einer ambulanten geriatrischen Rehabilitation sinnvoll.

Aus den weiteren Ergebnissen lässt sich zudem ableiten, in welches Umfeld die Patient*innen der akutstationären geriatrischen Rehabilitation oftmals entlassen werden. Aufgrund der Struktur der Entlassbriefe bleibt allerdings unklar, ob die Versorgung lediglich über die hausärztliche Behandlung erfolgte oder zudem ein ambulanter Pflegedienst implementiert war oder wurde.

Abschließend wurden im Forschungsvorhaben die Ergebnisse der standardisierten Assessments analysiert. Allerdings lässt sich die Effektivität einer solch komplexen Intervention wie der stationären geriatrischen Rehabilitation nicht allein anhand der untersuchten Assessmentergebnisse beschreiben, sodass es sich an dieser Stelle nur um einen Betrachtungsausschnitt handelt. In einigen wenigen Fällen (wie zuvor im Abschnitt „Auswertung von Assessmentergebnissen“ erwähnt) zeigte sich trotz der stationären Behandlung auch eine Verschlechterung (um bis zu 35 %), was zu Ausreißern in der Darstellung des Barthel-Index führt. Interessant wäre an dieser Stelle zu untersuchen, ob diese Gruppe von Patient*innen mit der Gruppe der Patient*innen korrelierte, die (vielleicht aufgrund akuter anderer Erkrankung) in andere Kliniken verlegt wurden – diesem Aspekt sollte prinzipiell weiter nachgegangen werden.

### Methodenkritik

Methodisch weist die beschriebene Vorgehensweise einige Limitationen auf. Sie ist statisch und speziell auf die vorliegenden 372 Entlassbriefe zugeschnitten, und es ist zu erwarten, dass für die Analyze von Briefen anderer Einrichtungen einige Anpassungen vorgenommen werden müssten. Eine Erweiterung um Indikatoren ist jedoch vorstellbar und jederzeit möglich.

Zudem können derzeit ausschließlich die explizit definierten Informationen extrahiert werden. Zu prüfen ist, inwiefern sich große Sprachmodelle zu einer spontanen Analyse nutzen lassen. Aufgrund von Datenschutzaspekten wird dabei auf offene Modelle zurückgegriffen, beispielsweise Mistral [6] oder deutsche Varianten wie z. B. Phoenix [9]. Um die Verlässlichkeit zu erhöhen, sollten dabei Prompt-Engineering-Techniken wie Retrieval-Augmented Generation [8] genutzt werden. Ein solcher Ansatz hätte die Vorteile, Sprachmodelle unabhängiger vom tatsächlichen Aufbau des Briefes zu machen, Kontext miteinzubeziehen und eine Erweiterbarkeit durch Laien zu ermöglichen, indem flexibel weitere Fragen für den gezielten Abruf von Informationen („Retrieval“) hinzugefügt werden können. Ein solcher Ansatz wäre unabhängiger von der Struktur des eigentlichen Briefes. Zudem könnten Informationen ausgelesen werden, die nur implizit im Brief enthalten sind. Große Sprachmodelle sind jedoch mit dem Risiko behaftet, Informationen zu fantasieren. Daher ist die Erstellung eines Testdatensatzes erforderlich, um die Verlässlichkeit des Systems mit Zahlen zu belegen. Darüber hinaus besteht die Problematik, dass ein derartiges System einen hohen Bedarf an Rechenleistung aufweist. Dies kann einerseits zu Kosten führen und stellt andererseits eine potenzielle Problematik in Bezug auf den Datenschutz dar. Die Implementierung eines spezialisierten Trainingsprogramms würde die Durchführung mittels kleinerer Modelle ermöglichen. Für die Generierung eines annotierten Datensatzes ist jedoch ein signifikanter personeller Aufwand erforderlich.

Ferner bleibt zu betonen, dass es in der aktuellen Variante, insbesondere bei der Auswertung der Diagnosekategorien, zu Ungenauigkeiten kommt. Einerseits ist die Liste der Schlag- und Teilworte noch nicht vollständig bzw. variabel und könnte ergänzt werden. Andererseits werden z. T. und nicht unbedingt konsistent Abkürzungen genutzt, die laufend aktualisiert werden müssten, wie beispielsweise HWI für Harnwegsinfektion oder Hinterwandinfarkt. Vor diesem Hintergrund ist insbesondere der Teil der Schlagwortanalyse kritisch und stetig zu reflektieren. Da dies jedoch alle Kategorien gleichermaßen betrifft, und unter den Briefen lediglich 3 Exemplare identifiziert werden konnten, die sich keiner Kategorie zuordnen ließen, sind die relativen Häufigkeiten belastbar. Dennoch bietet gerade dieser Teil Raum für Verbesserungen in Form von Erweiterung der Begriffslisten und Schärfung der entwickelten Kategorien.

## Zusammenfassung und Fazit

Anhand des vorgestellten Projekts des Bielefelder Reallabors Geriatrie lässt sich zunächst erkennen, wie ein inter- sowie transdisziplinärer Forschungs- und Entwicklungsansatz gelebte Praxis zu werden vermag. Mit dem Ziel, die medizinische, pflegerische und therapeutische Gesundheitsversorgung an der Schnittstelle zur Technologieentwicklung zu verbessern und Potenziale des technologischen Fortschritts praxistauglich für die Qualitätssicherung im Bereich des Entlassmanagements geriatrischer Patient*innen einzusetzen, wurde der Entlassbriefanalyzer entwickelt und erprobt. Es wurde gezeigt, dass Daten aus Entlassbriefen auch innerhalb der sensiblen IT-Infrastruktur eines Krankenhauses aus vergleichsweisen großen Datenmengen niedrigschwellig extrahiert werden können, um Hinweise zu Indikationen, Therapieverläufen und die Allokation der Patient*innen zu erhalten. Insbesondere die Zuordnung in die entwickelten Diagnosekategorien ist an der Schnittstelle zwischen stationärer sowie ambulanter Versorgung von hoher Relevanz, da auf diese Weise eine Grundlage geschaffen wird, um die Bedarfe geriatrischer Patient*innen in anschließende Versorgungsformen zu klären und eine möglichst gute Versorgung sicherzustellen.

## Fazit für die Praxis

- Der Entlassbriefanalyzer ist geeignet, große Datenmengen innerhalb der sensiblen krankenhausseitigen IT-Infrastruktur zu verarbeiten.
- Die Analyse ermöglicht, vorab festgelegte Parameter aus Entlassbriefen zuverlässig auszulesen, wobei die Möglichkeiten durch den Einsatz großer Sprachmodelle zukünftig noch erweiterbar sind.
- Die Erstellung von Diagnosekategorien und die Zuordnung von Patient*innen in diese können genutzt werden, um jetzige und zukünftige Bedarfe von (geriatrischen) Patient*innen und die jeweiligen Größenordnungen einzuschätzen sowie die Versorgung besser kanalisieren zu können.

## Supplementary Information

In dem Zusatzmaterial online findet sich eine Darstellung der erhobenen Parameter sowie eine Übersicht der Verschlagwortung der Diagnose-Kategorien sowie Allokation.

## Data Availability

Die in dieser Studie erhobenen Datensätze können auf begründete Anfrage beim Korrespondenzautor angefordert werden.
