# Supplementary material for: Software-supported analysis of geriatric discharge letters: Potential of technology-driven data analysis in quality and discharge management at the transition to outpatient care
Source: Z Gerontol Geriatr. 2025 Aug 18;59(3):231–7. [Article in German] doi: 10.1007/s00391-025-02478-6 (PMC13109220; doi:10.1007/s00391-025-02478-6)
Supplement: Supplementary file 1 — In dem Zusatzmaterial online findet sich eine Darstellung der erhobenen Parameter sowie eine Übersicht der Verschlagwortung der Diagnose-Kategorien sowie Allokation. [file 391_2025_2478_MOESM1_ESM.docx]

Online Supplement

| Tab. 1 Erhobene Parameter | |
| --- | --- |
| Zahlenwerte | Alter, Barthel-Index, Timed up an go, Tinetti (gesamt), Tinetti Banalce, Tinetti Gang, Gehstrecke, Nutritional Risk Score, Geriatrische Depressionsskala, MMST, Uhrenergänzungstest |
| Freitext | Hilfsmittel, Diagnosen, Geschlecht |
| Diagnose-Kategorien | sturzbedingte, alterstraumatologische Indikation; Schlaganfall; Dekompensierte Herzinsuffizienz; neurodegenerative Grunderkrankung mit »Eskalation«; Gebrechlichkeit multifaktorieller Genese |
| Allokationen | Häuslichkeit, Klinik, Rehabilitation, Seniorenheim, Kurzzeitpflege, Betreutes Wohnen |

| Tab. 2 Verschlagwortung der Diagnose-Kategorien und Allokation | | |
| --- | --- | --- |
| Diagnose-Kategorie und Allokation | **Schlagworte** | **Teilworte** |
| sturzbedingte, alterstraumatologische Indikation | Fraktur, Subduralhämatom, subdurales Hämatom, Hämatom, Prellung, Wunde, Arthrose, Lockerung, Spinalkanalstenose | Fraktur, Hämatom, Prellung, traumatisch, degenerativ, Arthrose, Lockerung, Schmerz, algogen, SDH |
| Schlaganfall | Stroke, Schlaganfall, cerebrale Ischämie, zerebrale Ischämie, cerebraler Infarkt, zerebraler Infarkt, Apoplex, Insult, intracerebrale Blutung, intrazerebrale Blutung, intracranielle Blutung, intrakranielle Blutung | ICB |
| Dekompensierte Herzinsuffizienz | Herzinsuffizienz | Myokard, Herz, kardial |
| neurodegenerative Grunderkrankung mit »Eskalation« | Demenz, dementielle Entwicklung, Alzheimer, vaskuläre Demenz, Delir, delirant, Parkinson, Verwirrtheit, Amyotrophe Lateralsklerose, Multisystematrophie, cortikobasale Degeneration, progressive supranukleäre Blickparese | ALS, MSA, CBD, PSP |
| Gebrechlichkeit multifaktorieller Genese | frailty, Multimorbidität, Gangstörung, Gangunsicherheit, Sturzgefahr, multifaktoriell, Mobilität, Schmerz, Schmerzen, schmerzhaft, Infekt, Pneumonie, Lungenentzündung, Exsikkose, Sturzneigung, Carcinom, Karzinom | infekt, itis, Elektrolyt, natri, Sturz, Carcinom, Karzinom |
| Häuslichkeit | hausärztlich, häusliche, häuslichen, Hause, Häuslichkeit, ambulant, ambulante |  |
| Klinik | Klinik, stationär, Hospital, Krankenhaus, Station | Klinik |
| Rehabilitation | Reha, Rehabilitation, Rehabilitationszentrum, Sanatorium, Kurheim, Kurklinik, Rehabilitationsklinik, Erholungsstätte, RehaMaßnahme, RehaBehandlung, AHB, Anschlussheilbehandlung |  |
| Seniorenheim | Seniorenheim, Altenheim, Altenwohnheim, Seniorenresidenz, Pflegeheim, stationärem Rahmen, Heim, Wohneinrichtung, Pflegeeinrichtung |  |
| Kurzzeitpflege | Kurzzeitpflege, KZP |  |
| Betreutes Wohnen | betreutes |  |
